# Dendritic Cell-Secreted Lipocalin2 Induces CD8^+^ T-Cell Apoptosis, Contributes to T-Cell Priming and Leads to a T_H_1 Phenotype

**DOI:** 10.1371/journal.pone.0101881

**Published:** 2014-07-10

**Authors:** Melanie Floderer, Michaela Prchal-Murphy, Caterina Vizzardelli

**Affiliations:** 1 Laboratory of Tumour Immunology, St. Anna Children’s Cancer Research Institute, Vienna, Austria; 2 Institute of Pharmacology and Toxicology, Department for Biomedical Science, University of Veterinary Medicine Vienna, Vienna, Austria; University of Bergen, Norway

## Abstract

Lipocalin 2 (LCN2), which is highly expressed by dendritic cells (DCs) when treated with dexamethasone (Dex) and lipopolysaccharide (LPS), plays a key role in the defence against bacteria and is also involved in the autocrine apoptosis of T-cells. However, the function of LCN2 when secreted by DCs is unknown: this is a critical gap in our understanding of the regulation of innate and adaptive immune systems. Tolerance, stimulation and suppression are functions of DCs that facilitate the fine-tuning of the immune responses and which are possibly influenced by LCN2 secretion. We therefore examined the role of LCN2 in DC/T-cell interaction. WT or Lcn2^−/−^ bone marrow-derived DCs were stimulated with LPS or LPS+IFN-γ with and without Dex and subsequently co-cultured with T-cells from ovalbumin-specific TCR transgenic (OT-I and OT-II) mice. We found that CD8^+^ T-cell apoptosis was highly reduced when Lcn2^−/−^ DCs were compared with WT. An *in vivo* CTL assay, using LPS-treated DCs, showed diminished killing ability in mice that had received Lcn2^−/−^ DCs compared with WT DCs. As a consequence, we analysed T-cell proliferation and found that LCN2 participates in T-cell-priming in a dose-dependent manner and promotes a T_H_1 microenvironment. DC-secreted LCN2, whose function has previously been unknown, may in fact have an important role in regulating the balance between T_H_1 and T_H_2. Our results yield insights into DC-secreted LCN2 activity, which could play a pivotal role in cellular immune therapy and in regulating immune responses.

## Introduction

Dendritic cells (DCs) are professional antigen-presenting cells that coordinate innate and adaptive immune responses [1]. They are also the major source of cytokines, which can modulate effector cells. Because of these remarkable properties, DCs are valuable tools when developing vaccination strategies against tumours [2]. To improve their therapeutic use, it is important to understand their biology and how DCs regulate innate and adaptive immune responses in the tumour microenvironment [3], [4]. The results of recent studies suggest that DCs follow a multi-stage regulation program after encountering danger signals, which facilitates the fine-tuning of the immune response: tolerance maintenance, immune-stimulation and immune-suppression are time-dependent-maturation-functions of DCs [5]. Immature DCs patrol the body against pathogens and are active in endocytosis and in maintaining tolerance [2], [6]. However, when DCs encounter danger signals, such as pathogen-associated molecular patterns (PAMP) [7], [8], [9], they mature [10] with concomitantly increased expression of costimulatory molecules, cytokines [11], [12] and up-regulation of the major histocompatibility complex (MHC class I and II), all of which are immune-stimulators. Finally, during the late phase of DC maturation, DCs switch to a suppressive phenotype, which is characterized by the expression of molecules such as indoleamine 2,3-dioxygenase (IDO) [13], [14], [15], the soluble IL-2 receptor alpha molecules (sIL2RA, sCD25) [16] and IL-10 [17], [18], all immune-suppressors.

In earlier experiments, we identified high expression of LCN2 by DCs treated with dexamethasone (Dex) and LPS in a genome-wide expression analysis [19]. Glucocorticoids (GC) are immunosuppressive and anti-inflammatory drugs widely used to treat autoimmune diseases or allergies and to enhance or inhibit target-gene transcription. When treated with GC, DCs acquire a tolerogenic phenotype [20].

We wanted to determine the role of LCN2 when secreted by DCs. LCN2 is a small glycoprotein involved in a number of biological processes such as acute phase responses (APR) [21], [22], autocrine apoptosis of pro B-cells and IL-3-dependent bone marrow cells [23], [24], tumourigenesis [25], [26], [27], [28], and host defence against bacteria through the capture of iron-loaded siderophores [29], [30]. Because LCN2 is highly secreted after treatment with Dex and during late maturation, our hypothesis was that LCN2 is involved in the immune suppressive phase of DCs by inducing T-cell depletion. We therefore treated bone marrow-derived DCs with LPS and LPS+IFN-γ with and without Dex, to study the role of LCN2 in T-cell apoptosis in DC/T-cell interaction, using ovalbumin-specific TCR transgenic (OT-I and OT-II) mice. We then investigated *in vivo* T-cell suppression by means of a cytotoxic T-lymphocyte (CTL) killing assay using LPS treated Lcn2^−/−^ or WT DC immunisation. Finally, we monitored the *in vivo* microenvironment that had evolved from DC inoculation. Our results identified LCN2 as a previously unrecognized molecule involved in the regulation of the balance between T_H_1 and T_H_2. This has important implications for cellular immune therapy against cancer, allergies, and diseases such as type-1 diabetes.

## Materials and Methods

### Ethics Statement

All animal experiments were approved by the Institutional Review Board of the Medical University of Vienna and the Ministry of Sciences (BMWF-66.009/0323-11/3b/2012).

### Mice

Pathogen-free 8–12 week-old female C57BL/6 wild-type (WT) and Lcn2^−/−^ mice were used for experiments. Lcn2^−/−^ mice [29] were kindly provided by Akira, University Osaka, and were backcrossed for ten generations to a C57BL/6 background. Lcn2^−/−^ and C57BL/6 were bred and housed at the animal facility of the Medical University of Vienna. OT-I and OT-II transgenic mice were housed at the animal care unit of the Department of Pharmacology, Medical University of Vienna, Austria.

### Murine dendritic cells

DCs were derived from bone marrow cells [31]. Cells were resuspended in Iscove’s Modified Dulbecco’s Medium (IMDM from Gibco, Invitrogen) supplemented with 10% FCS (PAA, Laboratories GmbH, Pasching, Austria), 1 mM sodium pyruvate, 1 mM non-essential amino acids, 100 U/ml penicillin/streptomycin (Gibco), 50 µM β-mercaptoethanol (Sigma-Aldrich), 5 ng/ml recombinant murine IL-4 (eBioscence) and 3 ng/ml murine GM-CSF (BD, Pharmingen) and incubated at 37°C and 5% CO_2_ for 6 to 7 days.

### Stimulation and staining

Immature DCs (10^7^) were stimulated for 6 h with LPS (1 µg/ml *E. coli* strain O111:B4, Calbiotech Merck) or LPS in combination with IFN-γ (0.02 µg/ml, BD Pharmingen) with or without Dex (10^−8 ^M, Sigma-Aldrich) for 20 min before adding other reagents. To monitor maturation, DCs (10^5^) were stained with 5 µl of mix including anti-MHC-I, anti-MHC-II, anti-CD11b, anti-CD11c, anti-CD80, anti-CD83 and anti-CD86 (all from eBioscience, Austria).

T-cells were stained with anti-CD3, anti-CD4, anti-CD8, anti-CD25 and anti-Vα2 TCR for OT-I and OT-II mice (eBioscience). Cell viability was analysed with DAPI (Sigma-Aldrich). Apoptosis was measured with Annexin V (BD Pharmingen). CFSE (7 µM, Invitrogen) or Cell Proliferation Dye eFluor 670 (CPD, 5 µM, eBioscience) were used to detect proliferation. Flow cytometry was done on an LSR II (BD Pharmingen). Data were analysed by FlowJo (Version 9.6.2 Treestar). The difference in apoptosis induction was calculated using absolute cell number, determined with BD Trucount tubes.

This method is based on lyophilized pellet, containing a known number of fluorescent beads, which dissolves once the monoclonal antibody reagent is added. Absolute numbers (cells/µl) of positive cells in the sample are calculated following the equation: number of cell events/number of bead events x Trucount bead concentration.

### CD8^+^ and CD4^+^ T lymphocyte isolation

CD8^+^ and CD4^+^ T-cells were isolated from the spleens of OT-I or OT-II mice using MACS CD8^+^ or CD4^+^ Cell Isolation Kit (Miltenyi Biotech GmbH, Germany). T-cell purity of the enriched CD8^+^ or CD4^+^ was evaluated by flow cytometry.

### DC/T-cell co-culture

DCs were derived from the bone marrow of WT or Lcn2^−/−^ mice. After 6 days of culture, cell numbers were determined with BD Trucount tubes. Immature DCs were loaded with either 1 µg/ml SIINFEKL_257–264_-_,_ or OVA_323–339_-peptide (SIINFEKL_257–264,_ OVA_323–339,_ Bachem, Switzerland) for 40 min at 37°C, and then treated for 6 h with Dex alone, Dex 20 min prior LPS (Dex+LPS), Dex+LPS+IFN-γ, LPS+IFN-γ, LPS or untreated DCs as control. Treated and washed DCs (10^5^) were placed in culture with the same (1∶1, 10^5^ DC: 10^5^ T-cell) or higher (1∶5, 2×10^4^ DC: 10^5^ T-cell and 1∶10, 10^4^ DC: 10^5^ T-cell) amounts of either CD8^+^ or CD4^+^ T-cells isolated from the spleen of OT-I and OT-II mice. Before the co-culture, T-cells were stained with CFSE or CPD to later detect their proliferation by dilution of fluorescence. With some co-cultures, deferoxamine (Calbiochem) or recombinant Lcn2 (R&D) were also included in the culture medium.

### Protein and cytokine detection

LCN2 was detected using DuoSet ELISA (R&D Systems Europe) following the manufacturer’s protocol. IL-1α, IL-1β, IL-2, IL-4, IL-5, IL-6, IL-10, IL-12p70, IL-17, IL-22, TNF-α and IFN-γ were detected by FlowCytomix Multiple Analyte Detection System (eBioscence), Th1/Th2 10plex or single plex, by following the manufacturer’s protocol. Standard curves and cytokines amount were calculated using FlowCytomixPro software version 2.4.

### RNA isolation

RNA was isolated using Trizol (Sigma-Aldrich) and reverse transcribed to cDNA using the High Capacity cDNA Reverse Transcription Kit (Applied Biosystems, Life technologies, Austria).

### Quantitative Real Time PCR

Primers for Lcn2 (Mm 01324470_m1), Lcn2R (24p3receptor, Mm 00480680_m1), Megalin (Mm 01328171_m1) and β2-microglobulin (Mm 00437762_m1, Applied Biosystems) were used for RT-PCR. Amplifications were done using a 7500 Fast Real-Time PCR system cycler according to the manufacturer’s instructions. Gene expression was calculated following the equation 2^−ΔΔCT^ = [(C_T_ gene of interest – C_T_ internal control) sample A – (C_T_ gene of interest – C_T_ internal control) sample B] [32].

### *In vivo* Cytotoxic T-Lymphocytes assay

An *in vivo* CTL assay was performed [33], [34]. Bone marrow-derived WT and Lcn2^−/−^ DCs were loaded with SIINFEKL_257–264_- and OVA_323–339_-peptides and then stimulated with LPS for 6 h. Afterwards, the cells were washed, counted and resuspended in PBS (1x) to reach a final concentration of 5×10^7^/ml to inject 200 µl s.c. Five mice received Lcn2^−/−^ DCs, five received WT DCs and five, as control, received just PBS (1x). After 3 days, syngeneic splenocyte-target cells were pulsed for 1 hour at 37°C either with 10 µg/ml SIINFEKL_257–264_ or 10 µg/ml OVA_323–339_ or 10 µg/ml TRP-2_158–165_ (Tyrosine-related protein 2, L-dopachrome tautomerase, an irrelevant peptide, Bachem) and labelled with different concentrations of CFSE (2.5 µM for SIINFEKL, 0.25 µM for OVA and 0.025 µM for TRP-2 target-cells). 100 µl of the 1∶1:1 (10^8^/ml) mixed target-cell suspension were injected i.v. in DC-immunised or control mice. After 6 and 18 h, draining lymph nodes and spleen were taken for single cell isolation. Lymphocytes were acquired by flow cytometry and analysed by FlowJo. Splenocytes were used for intracellular staining of cytokines and transcription factors. Specific lysis was calculated as 1– (% CFSE^hi/^% CFSE^lo^)×100. Results are expressed as mean ± SEM.

### Intracellular staining

Five replicates of splenocytes (10^5^) were plated in 200 µl of medium supplemented with 0.05 µg/ml PMA (Sigma-Aldrich), 0.001 µg/ml ionomycin calcium salt (Sigma-Aldrich) and 0.2 µl BD GolgiPlug (brefeldin A, BD Pharmingen). As control, the cells were stimulated with PMA and Ionomycin without GolgiPlug. After 4 h of incubation at 37°C and 5% CO_2_, 100 µl of the supernatant was frozen at −20°C for ELISA. The cells were first stained with anti-CD8α and anti-CD4 before following the instruction for cytokines’ intracellular staining with a mix of anti-IL-2, anti-IL-4, anti-IL-10, anti-IL-17, anti-IL-22 and anti-IFN-γ, or intracellular staining for transcription factors with a mix of anti-Foxp3, anti-T-bet, anti-ROR-γt and anti-Gata-3 (eBioscience). The staining of intracellular cytokines and enzymes of CD8^+^ T-cells was done with a mix of anti-IL-2, anti-IFN-γ, anti-granzyme B and anti-perforin (reagents and isotypes from eBioscience). For FACS analysis, cells were resuspended in 50 µl PBS (1x).

### ELISPOT

Elispot analysis was performed after 3 days of DC/T-cell co-culture. After that CD4^+^/OT-II T-cells were counted and plated again over night at 37°C in a 96-well/ELISpot-plate (Cellular Technology Limited, CTL, ImmunoSpot, Cleveland, USA), with 6 h-LPS-treated DC-OVA, just OVA or TRP as controls, and PMA+Ionomycin. To monitor unspecific signals and background, T-cells, DC-OVA, DC-TRP, Lcn2^−/−^ DCs and WT DCs, were also co-cultured. We used ELISpot plates (Millipore, Massachusetts, USA) for IFN-γ, IL-10 and IL-17A (from Mabtech, Sweden), following the ImmunoSpot protocol and recommendations. Spots were counted using Immunospot 5.0 software. Histograms are the results of the spots from T-cell+WT DC-OVA or T-cell+Lcn2^−/−^ DC-OVA calculated after subtracting the spots of the controls.

### Statistical Analysis

Data are expressed as mean ± SEM. Two groups were compared using unpaired student’s t-test and multiple comparisons using two-way ANOVA followed by Bonferroni post-tests. A p value <0.05 was considered as statistically significant (*p<0.05; **p<0.01; ***p<0.001; ****p<0.0001). The obtained data were analysed by GraphPad Prism, version 5.0.

## Results

### LCN2 is not affecting DC maturation

**Figure 1:**
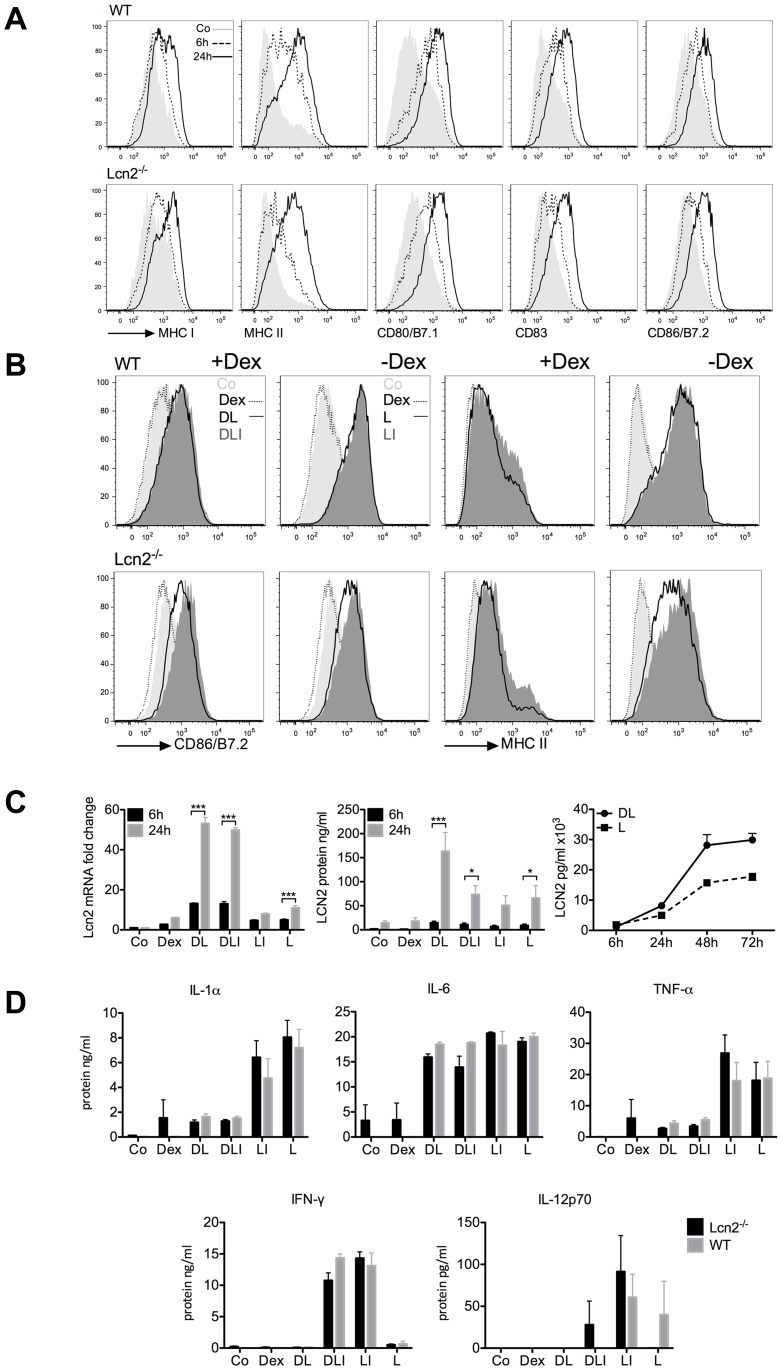
Characterisation of WT and Lcn2^−/−^ DC maturation. (A) Bone marrow-derived DCs were stained for major histocompatibility complex MHC class I and II, co-stimulatory molecules CD80/B7.1, CD86/B7.2 and the maturation marker CD83 after LPS treatment for 6 and 24 h. Negative control (Co). (B) Comparison of WT and Lcn2^−/−^ bone marrow-derived DCs treated for 24 h with Dexamethasone (Dex) added 20 min prior LPS (DL) and LPS+IFN-γ (DLI), or LPS (L), LPS+IFN-γ (LI), and analysed by FACS after CD86/B7.2 and MHC Class II staining. Negative control (Co). (C) Expression of Lcn2 mRNA and protein in DCs treated for 6 and 24 h with Dex, Dex+LPS (DL), Dex+LPS+IFN-γ (DLI), LPS+IFN-γ (LI), LPS alone (L) or on the left without any treatment (Co). The kinetic of LCN2 secretion was performed up to 72 h in DCs stimulated with DL and L and analysed by ELISA. The difference in the amount was due to the distinct amount of plated DCs. The two groups were analysed by ANOVA. P value is *p<0.05, ***p<0.001. (D) Proinflammatory cytokines IL-1α, IL-6, TNF-α, IFN-γ and IL-12p70 were measured in supernatant of DCs treated for 6 h with Dex, DL, DLI, LI, and L or on the left without any treatment (Co) as control, and quantified by Cytomix. IFN-γ identified what was previously added. These data are pooled from 6 independent experiments performed in triplicates and shown as mean ± SEM.

LCN2 is widely expressed by epithelial cells, fibroblasts, macrophages, T-cells [23] and DCs [19], and it is also present in the uterus during involution, a period of extensive apoptosis [22]. To find out whether LCN2, when secreted by DCs, participates in tolerance induction by T-cell clonal deletion, we first monitored and compared WT and Lcn2^−/−^ DC maturation and then confirmed LCN2 mRNA and protein expression in bone marrow-derived DCs [31] by using quantitative real-time PCR and ELISA. Immature DCs were treated with LPS and LPS+IFN-γ, with or without Dex, and maturation was monitored by flow cytometry. Costimulatory molecules and the MHC class I and II increased over time after LPS stimulation. We observed a delayed up-regulation of MHC class II during the first 6 hours in the Lcn2^−/−^ DCs, which reached a similar level in WT and Lcn2^−/−^ DCs after 24 hours (figure 1A). Thus, LCN2 does not disturb DC maturation and antigen presentation. Maturation was reduced by pre-treatment with Dex (figure 1B) as shown by Matyszak *et al.* [35]. IFN-γ was used to mimic human monocyte-derived DCs that had been stimulated with LPS+IFN-γ to increase the priming of T-cells [11], [36]. Interestingly, when Dex was added prior to LPS+IFN-γ, human monocyte-derived DCs did not fully mature (figure S1). This is noteworthy because DC maturation delay is the first step in immune suppression, and GC, like Dex, are used in clinical trials for the treatment of cancer, allergies and autoimmune diseases. In mice, as expected [19], Lcn2 was highly transcribed by cells that had been pre-treated with Dex (figure 1C). The increased expression after Dex+LPS stimulation has been confirmed in other cell types, as, *e.g*, in deactivated macrophages from the lung [37]. Secreted LCN2 levels steadily rose over time (figure 1C). LCN2 was also found when DCs were treated only with LPS or LPS+IFN-γ (figure 1C). After 6 hours of treatment, we monitored the increase in secreted levels of the proinflammatory cytokines IL-1α, IL-6, IL-12, TNF-α and IFN-γ. Although differences were observed between WT and Lcn2^−/−^ DC in the secreted levels of these cytokines (figure 1D), they were not statistically relevant. This result proved that Lcn2^−/−^ DC function and secretion are comparable to WT. Our data show that LCN2 is expressed by bone marrow-derived DCs; it is highly secreted when Dex is added prior to other stimulants and the secreted level increases over time. As with mouse DCs, human monocyte-derived DCs did not undergo full maturation with Dex. To explain this observation, we hypothesize that LCN2, which is highly expressed during the late phase of DC maturation, promotes the suppressive phenotype of DCs.

### The absence of LCN2 results in reduced CD8^+^ T-cell apoptosis

**Figure 2:**
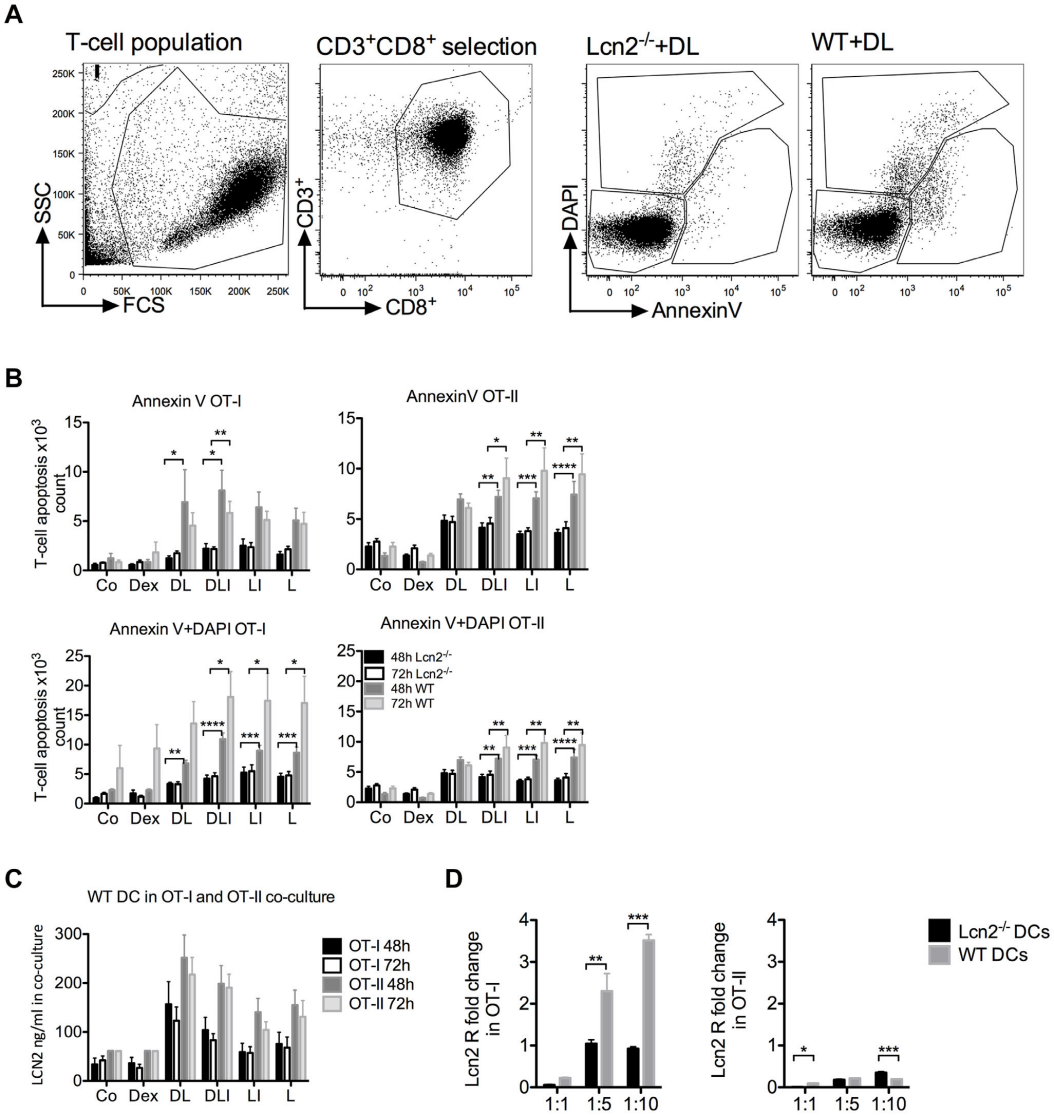
WT and Lcn2^−/−^ DC/T-cell co-culture and analysis of CD8^+^ T-cell apoptosis. T-cells were analysed after DC/T-cell co-culture by flow cytometry. WT and Lcn2^−/−^ DCs were treated for 6 h with Dex, Dex+LPS (DL), Dex+LPS+IFN-γ (DLI), LPS+IFN-γ (LI), LPS alone (L) or on the left without any treatment (Co), and co-cultured with OT-I or OT-II T-cells for 48 and 72 h. (A) Gating strategy, FACS analysis was done with cells positive for CD3^+^CD8^+^ or CD3^+^CD4^+^ fluorescence and the output data were divided in 3 main groups: alive, Annexin V and DAPI to distinguish early and late apoptosis. (B) Early and late apoptosis of OT-I or OT-II T-cells after 48 and 72 h. OT-I T-cells, 1240, underwent early apoptosis in DL-treated Lcn2^−/−^ DCs compared with WT DCs co-culture, 6920, p value <0.05. In the same condition, the comparison of 4829 and 6958 OT-II T-cells was not statistically relevant. Absolute cell numbers were determined with Trucount (BD). The two groups were analysed by ANOVA followed by Bonferroni correction for multiple comparison test (*p<0.05, **p<0.01, ***p<0.001, ****p<0.0001). P value refers to the comparison at 48 or 72 h. (C) LCN2 protein expression measured by ELISA in supernatant from treated WT DC/OT-I or treated WT DC/OT-II T-cell co-cultures after 48 and 72 h incubation. These data were pooled from 3 independent experiments performed in triplicate per OT condition and are shown as mean ± SEM. (D) Quantitative PCR (TaqMan) analysis of LCN2 receptor, Lcn2R (Slc22A17) expression in OT-I and OT-II T-cells after 72 h DC/T-cell co-cultures at 1∶1, 1∶5, 1∶10 ratio. Black bars represent T-cells co-cultured with 6 h-LPS-treated Lcn2^−/−^ DCs, grey bars represent the co-culture with 6 h-LPS-treated WT DCs. The two groups were analysed by ANOVA. P value is *p<0.05, **p<0.01, ***p<0.001.

LCN2 induces autocrine apoptosis in activated T-cells and in pro B-cells when IL-3 is withdrawn from the culture medium [23]. In order to investigate the induction of T-cell apoptosis mediated by the LCN2 secreted by DCs, we set up co-cultures with WT or Lcn2^−/−^ DCs and T-cells from OT-I or OT-II transgenic mice. OVA-loaded DCs were differently stimulated for 6 hours in order to obtain distinct amounts of secreted LCN2. The DC/T-cell cultures were analysed after 48 and 72 hours. T-cells were stained with CD3^+^, CD4^+^ or CD8^+^ antibodies, and analysed for apoptosis using Annexin V and DAPI to identify dead cells (figure 2A). During the first 48 hours, CD8^+^ T-cells underwent apoptosis when the DCs were treated with Dex, which resulted in an increase in secreted LCN2 (figure 1C). However, T-cell apoptosis was highly reduced in co-cultures with Lcn2^−/−^ DCs. After 48 hours we observed higher apoptosis in the presence of LCN2 for OT-I, while the difference was not significant for OT-II. CD4^+^ T-cells did not appear to be affected by apoptosis in the Dex+LPS (DL) condition. After 72 hours the effect of Dex was less evident (figure 2B). Our results indicate that induction of apoptosis is reduced when T-cells are co-cultured with Lcn2^−/−^, compared with WT DCs, and the response differs between OT-I and OT-II T-cells. We then measured the amount of secreted LCN2 in the co-cultures with WT and Lcn2^−/−^ DCs (figure 2C, figure S2). We found that in OT-I, LCN2 expression was less than in OT-II, and that CD8^+^ T-cells seemed to take up higher amounts of LCN2 (figure 2C). We therefore analysed the expression of Lcn2 receptors in OT-I and OT-II cells by qRT-PCR, Slc22A17 (known as Lcn2R) and Megalin (not found in our system). We found that CD8^+^ T-cells expressed Lcn2R but CD4^+^ T-cells did not (figure 2D). We then decided to analyse if the pro-apoptotic protein Bim [24] was differently expressed, and hence stimulated CD8^+^ and CD4^+^ T-cells with recombinant LCN2 and dynabeads (CD3CD28). We found no difference in Bim expression between CD8^+^ and CD4^+^ T-cells (data not shown).

Taken together, there is a correlation between high LCN2 expression and elevated apoptosis for CD8^+^ T-cells but not for CD4^+^ T-cells. We concluded that the apoptosis response is T-cell-specific, and one possible explanation for this is the different receptor expression levels in T-cells.

### LCN2 DCs do not impair the CD8 T-cell response

**Figure 3:**
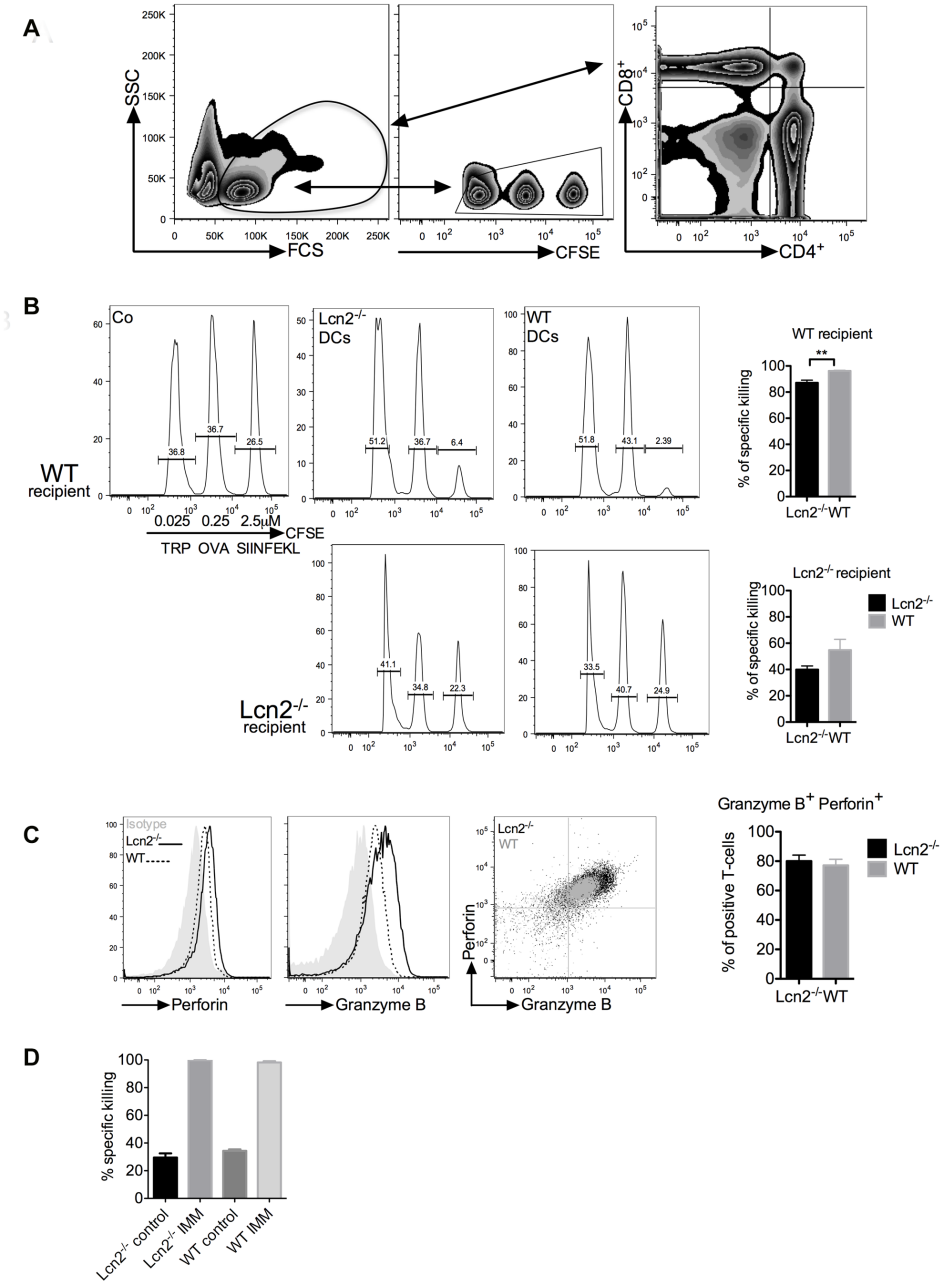
CTL assay with DC and peptide immunisation. (A) Gating strategy of lymph nodes, splenocytes were selected for CFSE staining and analysed. On the right, CD8^+^ and CD4^+^ T-cells in lymph nodes. (B) Analysis of the splenocytes in immunised WT or Lcn2^−/−^ recipient mice. Cells were selected based on the CFSE staining. On the left, the control group received just PBS, in the middle mice immunised with 6 h-LPS-treated Lcn2^−/−^ DCs and on the right, mice immunised with 6 h-LPS-treated WT DCs. Histograms show the specific killing effects using Lcn2^−/−^ or WT DC immunisation. These data show the most representative of pooled mice (5 mice/group) performed 3 independent times and shown as mean ± SEM, p value is **p<0.01. (C) FACS analysis of perforin and granzyme B intracellular staining in OT-I cells after 72 h of WT or Lcn2^−/−^ DC/T-cell co-culture. DCs were pre-treated for 6 h with LPS. Histograms show the percentage of positive cells in the co-culture. (D) Comparison of *in vivo* CTL assays in WT and Lcn2^−/−^ recipient mice immunized with SIINFEKL_257–264_ and CpG peptides for 7 days. These data show the most representative experiment of pooled mice (5 mice/group) performed 3 independent times.

**Figure 4:**
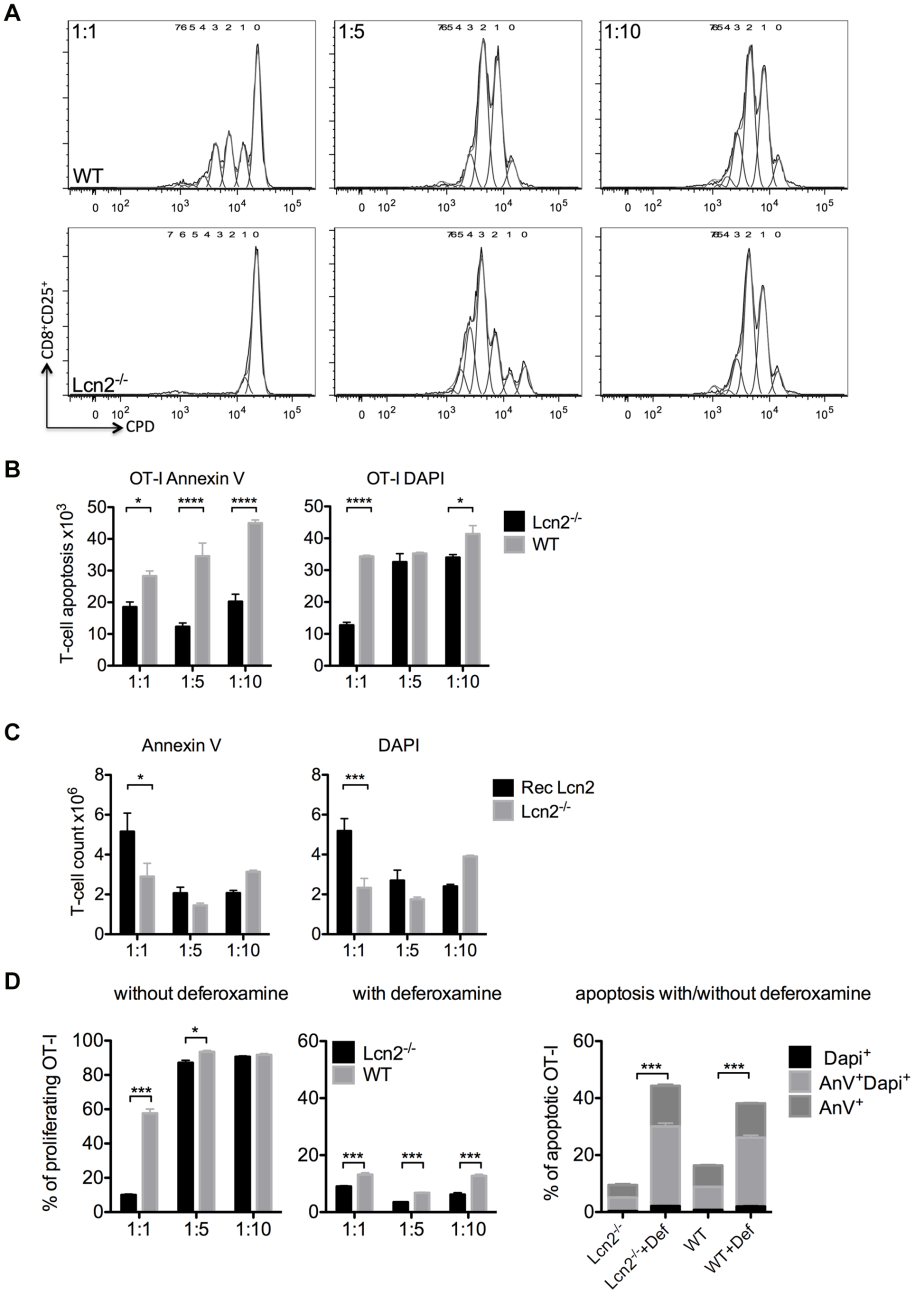
T-cell priming in DC/T-cell co-culture. WT and Lcn2^−/−^ DCs were pre-treated for 6 h with LPS and then co-cultured with T-cells. The extent of proliferation was calculated after 72-culture from the decrease in fluorescence per cell of the cell proliferation dye (CPD). (A) Histograms represent CD8^+^ T-cell divisions. Activated T-cells were stained and gated on cells positive for CD8^+^CD25^+^. Generation 0 was the undivided population initially labelled with CPD and all other generations were the result of the sequentially halved CPD fluorescence. The results were analysed by the proliferation tool of FlowJo. (B) Analysis of the T-cell apoptosis at 1∶1 (10^5^∶10^5^), 1∶5 (2×10^4^∶10^5^) and 1∶10 (10^4^∶10^5^) DC/T-cell ratios. T-cells (OT-I) were co-cultured with 6 h-LPS-treated Lcn2^−/−^ DCs (black bars) or 6 h-LPS-treated WT DCs (grey bars). Five independent experiments were performed in triplicate. Mean ± SEM, *p<0.05, ****p<0.0001. (C) Analysis of T-cell apoptosis after adding Recombinant Lcn2 (Rec Lcn2) in DC/T-cell co-culture. Lcn2^−/−^ DCs, pre-treated with LPS for 6 h, were co-cultured 72 h with T-cells in medium supplemented with Rec Lcn2. Five independent experiments were performed in triplicate. Mean ± SEM, *p<0.05, ***p<0.001. (D) Proliferation and apoptosis of T-cells (OT-I) was determined after co-culture in the absence or presence of desferrioxamine (1∶1, 1∶5 and 1∶10 ratio) for 72 h with DCs that had been previously incubated for 6 h with LPS. The two groups were analysed by ANOVA and shown as mean ± SEM, *p<0.05, ***p<0.001.

**Figure 5:**
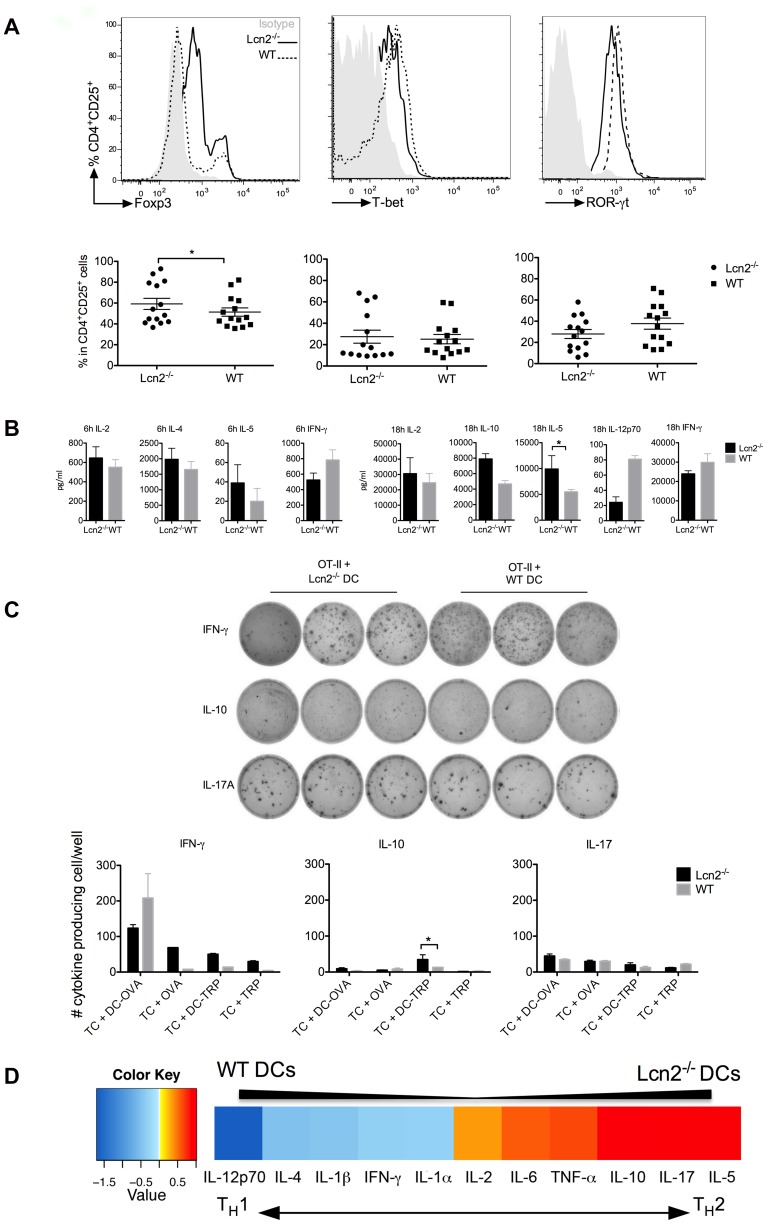
Analysis of the cytokine microenvironment after DC immunisation. (A) Histograms from FACS analysis of transcription factors expressed in CD4^+^ T-cells. Grey histograms are T-cells stained with isotype control, the dashed line represents cells from mice that had received WT DCs, the continuous line represents the mice injected with Lcn2^−/−^ DCs. The graphs below show the percentage of CD4^+^CD25^+^ T-cells used for statistical analysis. Fourteen mice were analysed using the student’s t-test, p value is *p<0.05. (B) The expression levels of cytokines in the spleens of immunised mice were measured 6 and 18 h after target-cell inoculation. The cytokine levels are shown for WT mice that had received 6 h-LPS-treated Lcn2^−/−^ DCs (black bars) or 6 h-LPS-treated WT DCs (grey bars). These data, shown as mean ± SEM, are the most consistent results of experiments on pooled mice (5 mice/group) from 3 independently performed experiments. (C) IFN-γ, IL-10 and IL-17A expression in CD4^+^ T-cells measured by ELISPOT. Pooled spots from triplicates of OT-II plus Lcn2^−/−^ or WT DC-OVA calculated after subtracting number of spots per well of the controls. Histograms show mean ± SEM and they were analysed using student’s t-test, p value is *p<0.05. (D) Ranking of cytokines according to the difference of their expression after DC immunisation. The ratio of expression between mice receiving Lcn2^−/−^ DCs or WT DCs inoculation was calculated. Then the cytokines were ranked based on these differences. For visualization the data were log2 transformed. The extremities represent the highest difference in secretion (mice receiving Lcn2^−/−^ DCs secreted more IL-5 than mice that received WT DCs, value 0.5). The left side represents reduced secretion by Lcn2^−/−^ DCs compared to WT DCs, value −1.5. The ranking orders distinguish two distinct environments after DC immunisation: a T_H_1 after WT DCs and a T_H_2 after Lcn2^−/−^ DC immunisation.

To test whether LCN2 affects the *in vivo* CD8^+^ T-cell response, we set up a cytotoxic T-lymphocyte killing assay (CTL), using DC immunisation. OVA-loaded DCs were stimulated for 6 hours with LPS and delivered s.c. to syngeneic WT mice. Dex was not used because, based on our protocol, it prevents the up-regulation of CCR7 cytokine, which is responsible for the DC migration to lymph nodes [19]; this is in contrast to Anderson *et al.* and Volchenkov *et al.* who used another treatment condition [38], [39]. Three days after immunisation, mice received splenocyte-target cells loaded with distinct OVA-peptides in combination with different concentrations of fluorochrome. The draining lymph nodes were harvested after 6 and 18 hours and CFSE positive cells were quantified (figure 3A). Three peaks were detected in the control mice. Mice that had received DC immunisation showed a significant reduction of the SIINFEKL peak (Lcn2^−/−^ DCs 6.4%, WT DCs 2.4% remaining cells, figure 3B). We observed a reduced killing response in mice receiving Lcn2^−/−^ DCs (87% ±2.0%) compared with WT DCs (96% ±0.5%, p<0.01). The WT DC immunisation induced a higher cytotoxic response. Based on these results, we hypothesized that immunisation with Lcn2^−/−^ DCs resulted in either improper priming of T-cells, or the development of CD4^+^ regulatory T-cells. To establish which hypothesis was correct we analysed T-cell proliferation and microenvironment (figure 4 and figure 5).

When the experiment was repeated in Lcn2^−/−^ mice, the killing response was reduced considerably as compared to WT mice, going from 87% to 40% (±2.8) with Lcn2^−/−^ DCs, and from 96% to 55% (±8.1) with WT DCs (figure 3B bottom). Although the trends were the same as for WT mice, the functionality and lytic ability were significantly reduced in Lcn2^−/−^ mice (p<0.0001 figure S3). *In vitro* intracellular staining for granzyme B and perforin of OT-I T-cells co-cultured with WT or Lcn2^−/−^ DCs showed a trend not statistically relevant (figure 3C), indicating that LCN2 probably does not affect the cytotoxic ability of CD8^+^ T-cells. To test this conclusion for the *in vivo* condition, we used SIINFEKL_257–264_ as peptide together with the adjuvant CpG [40], [41] to immunise WT and Lcn2^−/−^ mice for 7 days before inoculating target cells and 18 hours later measuring the killing effect. No difference was found between the WT and Lcn2^−/−^ responses, antigen presentation and cytotoxic activity were not damaged by the absence of LCN2 (figure 3D). In summary, LCN2 does not affect the killing function of CD8^+^ T-cells. The large difference between WT and Lcn2^−/−^ recipient mice led us to speculate that other cell types mediate the response we observed with DC immunisation.

### LCN2 expressing DCs promote T-cell proliferation

LCN2 is known to be an iron-binding protein that can deliver iron to cells when LCN2, bound to the siderophore-iron complex (holo-Lcn2), is internalized. When the iron complex is lacking (apo-Lcn2), LCN2 is internalized, chelates iron and reduces its intracellular concentration [24]. Since iron concentration is important for cell proliferation, we sought to verify the ability of Lcn2^−/−^ DCs to stimulate T-cells. We observed that WT DCs were able to activate both CD8^+^ and CD4^+^ T-cells, whereas Lcn2^−/−^ DCs showed a significantly impaired T-cell division at 1∶1 but not 1∶10 DC/T-cell ratio (figure 4A). This result might seem incongruous, but it is consistent with T-cells playing an active role in their own proliferation by producing IL-2 (data not shown). Independently of the cell ratio, apoptosis was always reduced when Lcn2^−/−^ DCs were used (figure 4B), but when recombinant LCN2 was added to Lcn2^−/−^ DC/T-cell co-culture, apoptosis increased again (figure 4C). A similar result was obtained using conditioned media from the WT DCs (figure S4). To find out whether T-cell proliferation depends on iron-transportation, we used a chelating agent, deferoxamine to remove iron from the co-culture and found that proliferation was highly reduced, while apoptosis increased, especially in the Lcn2^−/−^ DC/T-cell co-culture (figure 4D). We conclude that LCN2 contributes to the proliferation of T-cells, which could explain the reduced killing ability of the Lcn2^−/−^ in the *in vivo* CTL assay.

### DC immunisation alters the cytokine microenvironment

CD4^+^ T-cell differentiation depends on the stimulus, the cytokine milieu [42] and the strength of stimulation [43]. Therefore, we monitored the microenvironment after 3 days of DC immunisation and a further 6 and 18 hours of target-cell inoculation. The splenocytes were stained to detect transcription factors for regulatory T-cells (T_Reg_), T helper 1 (T_H_1) and T helper 17 (T_H_17), T helper 2 (T_H_2) and the expression of various cytokines. WT mice receiving Lcn2^−/−^ DCs showed slightly elevated levels of FoxP3 expression compared with mice receiving WT DCs (p<0.05), whereas T-bet, ROR-γt and Gata-3 showed no statistical change (figure 5A, figure S5). IL-2, IL-4, IL-5, IL-10, IL-12p70, as well as IFN-γ, were measured upon restimulation of the splenocytes with PMA and ionomycin (figure 5B). The mice receiving WT DC immunisation produced more IL-12p70 and IFN-γ after 6 and 18 hours of target-cell inoculation, indicating a tendency to induce a T_H_1 response. The IFN-γ secretion was also calculated using an Elispot assay to quantify the amount produced by the responding cells (figure 5C). Mice receiving Lcn2^−/−^ DCs showed increased expression of IL-2, IL-4, IL-5 and IL-10 (after 18 hours, figure 5B). Intracellular levels of IL-17 and IL-22 showed no statistical change after 6 hours (figure S6) but increased at 18 hours especially in mice that received Lcn2^−/−^ DCs, indicating a delayed response. In addition, we calculated the ratio of cytokine expression between WT and Lcn2^−/−^ DC immunisation (see figure 5D legend). The ranking order supports the observation that T-cell reprogramming was occurring, because the DCs induce two distinct environments: a T_H_1 environment in the case of WT DC inoculation and a T_H_2 milieu in the case of Lcn2^−/−^ DC immunisation. We concluded that, amongst other molecules, LCN2 promotes a T_H_1 response as opposed to the T_H_2 response that is favoured in its absence.

## Discussion

Professional antigen-presenting cells are pivotal in the regulation of innate and adaptive immune responses [1], [11], [12]. Specifically for this characteristic, DCs have been exploited as vaccines against cancer [44], [45]. The aim of DC vaccination is to induce tumour-specific effector T-cells to reduce tumour mass or induce prolonged and increased inflammation against tumour antigens. In this context, Nava et al., 2012, investigated the effect of DC vaccination against glioblastoma [46]. LCN2, an APR protein, may have a major role in DC/T-cell processes, given its abundant secretion by DCs after being treated with Dex and, subsequently, with LPS [19]. This secretion is not restricted to LPS stimulation but is also found with Dex in combination with IFN-γ. In our study, cells were treated with IFN-γ to mimic the human situation, where IFN-γ is used to trigger DC maturation, and we showed that LCN2 is highly expressed when Dex is added prior to other stimuli.

In a tumour milieu LCN2 is potentiated by proinflammatory cytokines Il-1β, TNF-α, and IL-17 [47] and is involved in the epithelial to mesenchymal transition (EMT) [48], [49]. LCN2, however, does have controversial roles: it is an APR [21], it plays a role in apoptosis [23] and it is bacteria-static by sequestering iron through its siderophores [29]. For all these reasons, it is important to understand the role of LCN2 when it is highly secreted by DCs.

We therefore monitored the kinetics of LCN2 secretion by the bone marrow-derived DCs. We found that DCs express LCN2 independently of subsets or culture conditions (GM-CSF alone or GM-CSF in combination with IL-4) [19], whereas mouse and human DCs efficiently secrete IL-12 (which is important for T-cell priming and T_H_1 response) only when previously exposed to IL-4 [50], [51]. The amount of secreted LCN2 is greatly increased by DCs incubated with Dex (DEX+LPS and DEX+LPS+IFN-γ). By contrast, IFN-γ does not seem to play a role in LCN2 release and, when used in combination with LPS, IFN-γ seems to reduce LCN2 secretion. This observation is consistent with the observed increased expression of LCN2 by macrophages infected with *Salmonella Typhimurium* when incubated with IFN-γ [52] but not when incubated with LPS. We thus conclude that LCN2 has a cell- and content-dependent function, which has also been well-documented in the mouse intestinal tumour model APCmin [53].

Before using WT and Lcn2^−/−^ DCs for *in vivo* experiments, we monitored *in vitro* the maturation and the expression of proinflammatory cytokines. The results showed that Lcn2^−/−^ DCs are as functional as WT DCs and that their secretion of proinflammatory cytokines is higher without Dex treatment. We then studied the role of LCN2 secreted by DCs in the induction of T-cell apoptosis. We showed that LCN2, when highly secreted by DCs, plays a role in promoting the apoptosis of T-cells in a manner that is CD8^+^ T-cell-specific. This might be due to the expression of the Lcn2 receptor on the T-cell surface [54]. LCN2 binds its Lcn2R and is internalized to deliver iron, which is important for the survival of T-cells. CD8^+^ T-cells expressed Lcn2R but CD4^+^ T-cells did not. We speculate that, together with iron-homeostasis, apoptosis occurs when there is an unequal distribution of LCN2 or Lcn2R. Most likely other molecules participate in apoptosis in the presence of Dex. Consistent with this proposed role of LCN2, we found that supplementing the Lcn2^−/−^ DC/T-cell co-culture with recombinant LCN2 resulted in increased T-cell apoptosis.

From our immunisation studies with DCs, we uncovered a subtle role of LCN2 in inducing both T-cell apoptosis and proliferation, and found that Lcn2^−/−^ DCs impaired T-cell priming ability. The cytokine microenvironment – as compared to Lcn2^−/−^ DC immunisation - also showed a distinct milieu when generated by WT DC. The WT DC inoculation induced a T_H_1 response through the enhanced secretion of IFN-γ and IL-12p70; conversely, Lcn2^−/−^ DCs triggered a T_H_2 response by releasing more IL-2, IL-4, IL-5 (which increased 10.000 fold within 12 hours), and IL-10 cytokines. Our data demonstrated that LCN2 plays a role in CD8^+^ T-cell apoptosis mediated by DCs, especially when pretreated with Dex+LPS. LCN2 was involved in T-cell priming after LPS stimulation in a ratio-dependent manner. DC immunisation was highly efficient: when using WT DCs, LCN2 contributed to an increase in the DCs’ anti-tumour effectiveness by inducing a T_H_1 microenvironment phenotype. Physiologically, this tended to develop towards a cell-mediated, rather than a humoral or antibody-mediated response. Clearly, it would be of great benefit to the cellular immune therapy treatment of cancer and diseases such as multiple sclerosis, type-1 diabetes, asthma and allergies to regulate the balance between T_H_1 and T_H_2 [43], [55]. While our data may be descriptive, they nonetheless demonstrate the importance of LCN2 in the DC/T-cell interaction. Further study using tetramer staining as well as on the LCN2 molecular mechanism should be done to unravel the dual role of LCN2 in T-cell commitment.

## Supporting Information

Figure S1 **FACS analysis of human monocytes-derived DC maturation after treatment with Dex+LPS+IFN-γ (DLI), LPS+IFN-γ (LI) and the negative control (Co) and stained with CD86.**
(TIF)

Figure S2 **LCN2 expression in Lcn2^−/−^ DC/OT-I and OT-II T-cell co-cultures, the protein amount is calculated in pg/ml, while in WT DC/OT-cell co-cultures it is in ng/ml.**
(TIF)

Figure S3 **Comparison of the mice killing ability, it is referred to the** ***in vivo*** **CTL assay with DC immunization.**
(TIF)

Figure S4 **T-cells in culture with conditioned medium from 24 h-LPS-treated WT and Lcn2^−/−^ DCs.**
(TIF)

Figure S5 **Intracellular staining for the transcription factor T helper 2 (Gata3).**
(TIF)

Figure S6 **Intracellular staining of cytokines in CD4^+^ T-cells.**
(TIF)

## References

[1] Banchereau J, Steinman RM (1998) Dendritic cells and the control of immunity. Nature 392: 245–252. 9521319 10.1038/32588

[2] Palucka K, Banchereau J (2013) Dendritic-cell-based therapeutic cancer vaccines. Immunity 39: 38–48. 23890062 10.1016/j.immuni.2013.07.004 PMC3788678

[3] Palucka K, Banchereau J (2012) Cancer immunotherapy via dendritic cells. Nat Rev Cancer 12: 265–277. 22437871 10.1038/nrc3258 PMC3433802

[4] Gabrilovich DI, Ostrand-Rosenberg S, Bronte V (2012) Coordinated regulation of myeloid cells by tumours. Nat Rev Immunol 12: 253–268. 22437938 10.1038/nri3175 PMC3587148

[5] Luger R, Valookaran S, Knapp N, Vizzardelli C, Dohnal AM, et al (2013) Toll-like receptor 4 engagement drives differentiation of human and murine dendritic cells from a pro- into an anti-inflammatory mode. PloS One 8: e54879. 23408948 10.1371/journal.pone.0054879 PMC3569454

[6] Zhou F, Ciric B, Zhang GX, Rostami A (2013) Immune tolerance induced by intravenous transfer of immature dendritic cells via up-regulating numbers of suppressive IL-10 (+) IFN-gamma (+)-producing CD4 (+) T cells. Immunol Res 56 (1): 1–8. 10.1007/s12026-012-8382-7 PMC3628929 23292714

[7] Medzhitov R, Janeway CA Jr (2002) Decoding the Patterns of Self and Nonself by the Innate Immune System. Science 296: 298–300. 11951031 10.1126/science.1068883

[8] Dunn GP, Bruce AT, Ikeda H, Old LJ, Schreiber RD (2002) Cancer immunoediting: from immunosurveillance to tumor escape. Nat Immunol 3: 991–998. 12407406 10.1038/ni1102-991

[9] Schreiber RD, Old LJ, Smyth MJ (2011) Cancer immunoediting: integrating immunity’s roles in cancer suppression and promotion. Science 331: 1565–1570. 21436444 10.1126/science.1203486

[10] Matzinger P (2002) The danger model: a renewed sense of self. Science 296: 301–305. 11951032 10.1126/science.1071059

[11] Felzmann T, Huttner KG, Breuer SK, Wimmer D, Ressmann G, et al (2005) Semi-mature IL-12 secreting dendritic cells present exogenous antigen to trigger cytolytic immune responses. Cancer Immunol Immunother 54: 769–780. 15647926 10.1007/s00262-004-0637-2 PMC11034250

[12] Granucci F, Vizzardelli C, Pavelka N, Feau S, Persico M, et al (2001) Inducible IL-2 production by dendritic cells revealed by global gene expression analysis. Nat Immunol 2: 882–888. 11526406 10.1038/ni0901-882

[13] Harden JL, Egilmez NK (2012) Indoleamine 2,3-dioxygenase and dendritic cell tolerogenicity. Immunol Invest 41: 738–764. 23017144 10.3109/08820139.2012.676122 PMC3645912

[14] Jurgens B, Hainz U, Fuchs D, Felzmann T, Heitger A (2009) Interferon-gamma-triggered indoleamine 2,3-dioxygenase competence in human monocyte-derived dendritic cells induces regulatory activity in allogeneic T cells. Blood 114: 3235–3243. 19625705 10.1182/blood-2008-12-195073

[15] Munn DH, Sharma MD, Lee JR, Jhaver KG, Johnson TS, et al (2002) Potential regulatory function of human dendritic cells expressing indoleamine 2,3-dioxygenase. Science 297: 1867–1870. 12228717 10.1126/science.1073514

[16] von Bergwelt-Baildon MS, Popov A, Saric T, Chemnitz J, Classen S, et al (2006) CD25 and indoleamine 2,3-dioxygenase are up-regulated by prostaglandin E2 and expressed by tumor-associated dendritic cells in vivo: additional mechanisms of T-cell inhibition. Blood 108: 228–237. 16522817 10.1182/blood-2005-08-3507

[17] Saraiva M, O’Garra A (2010) The regulation of IL-10 production by immune cells. Nat Rev Immunol 10 (3): 170–81. 10.1038/nri2711 20154735

[18] Zhou F, Ciric B, Li H, Yan Y, Li K, et al (2012) IL-10 deficiency blocks the ability of LPS to regulate expression of tolerance-related molecules on dendritic cells. Eur J Immunol 42: 1449–1458. 22622800 10.1002/eji.201141733 PMC3596886

[19] Vizzardelli C, Pavelka N, Luchini A, Zanoni I, Bendickson L, et al (2006) Effects of dexamethazone on LPS-induced activationand migration of mouse dendritic cells revealed by a genome-wide transcriptional analysis. Eur J Immunol 36: 1504–1515. 16708398 10.1002/eji.200535488

[20] Groux H, O’Garra A, Bigler M, Rouleau M, Antonenko S, et al (1997) A CD4+ T-cell subset inhibits antigen-specific T-cell responses and prevents colitis. Nature 389: 737–742. 9338786 10.1038/39614

[21] Liu Q, Nilsen-Hamilton M (1995) Identification of a new acute phase protein. J Biol Chem 270: 22565–22570. 7545679 10.1074/jbc.270.38.22565

[22] Ryon J, Bendickson L, Nilsen-Hamilton M (2002) High expression in involuting reproductive tissues of uterocalin/24p3, a lipocalin and acute phase protein. Biochem J 367: 271–277. 12067275 10.1042/BJ20020026 PMC1222854

[23] Devireddy LR, Teodoro JG, Richard FA, Green MR (2001) Induction of apoptosis by a secreted lipocalin that is transcriptionally regulated by IL-3 deprivation. Science 293: 829–834. 11486081 10.1126/science.1061075

[24] Devireddy LR, Gazin C, Zhu X, Green MR (2005) A cell-surface receptor for lipocalin 24p3 selectively mediates apoptosis and iron uptake. Cell 123: 1293–1305. 16377569 10.1016/j.cell.2005.10.027

[25] Lin H, Monaco G, Sun T, Ling X, Stephens C, et al (2005) Bcr-Abl-mediated suppression of normal hematopoiesis in leukemia. Oncogene 24: 3246–3256. 15735695 10.1038/sj.onc.1208500

[26] Leng X, Lin H, Ding T, Wang Y, Wu Y, et al (2008) Lipocalin 2 is required for BCR-ABL-induced tumorigenesis. Oncogene 27: 6110–6119. 18663364 10.1038/onc.2008.209 PMC2756829

[27] Berger T, Cheung CC, Elia AJ, Mak TW (2010) Disruption of the Lcn2 gene in mice suppresses primary mammary tumor formation but does not decrease lung metastasis. Proc Natl Acad Sci U S A 107: 2995–3000. 20133630 10.1073/pnas.1000101107 PMC2840296

[28] Yang J, Bielenberg DR, Rodig SJ, Doiron R, Clifton MC, et al (2009) Lipocalin 2 promotes breast cancer progression. Proc Natl Acad Sci U S A 106: 3913–3918. 19237579 10.1073/pnas.0810617106 PMC2656179

[29] Flo TH, Smith KD, Sato S, Rodriguez DJ, Holmes MA, et al (2004) Lipocalin 2 mediates an innate immune response to bacterial infection by sequestrating iron. Nature 432: 917–921. 15531878 10.1038/nature03104

[30] Devireddy LR, Hart DO, Goetz DH, Green MR A mammalian siderophore synthesized by an enzyme with a bacterial homolog involved in enterobactin production. Cell 141: 1006–1017. 10.1016/j.cell.2010.04.040 PMC2910436 20550936

[31] Hüttner KG, Breuer SK, Paul P, Majdic O, Heitger A, et al (2005) Generation of potent anti-tumor immunity in mice by interleukin-12-secreting dendritic cells. Cancer Immunol Immunother 54: 67–77. 15693141 10.1007/s00262-004-0571-3 PMC11034180

[32] Schmittgen TD, Livak KJ (2008) Analyzing real-time PCR data by the comparative C(T) method. Nat Protoc 3: 1101–1108. 18546601 10.1038/nprot.2008.73

[33] Schellack C, Prinz K, Egyed A, Fritz JH, Wittmann B, et al (2006) IC31, a novel adjuvant signaling via TLR9, induces potent cellular and humoral immune responses. Vaccine 24: 5461–5472. 16678312 10.1016/j.vaccine.2006.03.071

[34] Simma O, Zebedin E, Neugebauer N, Schellack C, Pilz A, et al (2009) Identification of an indispensable role for tyrosine kinase 2 in CTL-mediated tumor surveillance. Cancer Res 69: 203–211. 19118004 10.1158/0008-5472.CAN-08-1705

[35] Matyszak MK, Citterio S, Rescigno M, Ricciardi-Castagnoli P (2000) Differential effects of corticosteroids during different stages of dendritic cell maturation. Eur J Immunol 30: 1233–1242. 10760813 10.1002/(SICI)1521-4141(200004)30:4<1233::AID-IMMU1233>3.0.CO;2-F

[36] Felzmann T, Gadner H, Holter W (2002) Dendritic cells as adjuvants in antitumor immune therapy. Onkologie 25: 456–464. 12415201 10.1159/000067441

[37] Warszawska JM, Gawish R, Sharif O, Sigel S, Doninger B, et al (2013) Lipocalin 2 deactivates macrophages and worsens pneumococcal pneumonia outcomes. J Clin Invest 1; 123 (8): 3363–72. 10.1172/JCI67911 PMC3726165 23863624

[38] Anderson AE, Swan DJ, Sayers BL, Harry RA, Patterson AM, et al (2009) LPS activation is required for migratory activity and antigen presentation by tolerogenic dendritic cells. J Leukoc Biol 85: 243–250. 18971286 10.1189/jlb.0608374 PMC2700018

[39] Volchenkov R, Brun JG, Jonsson R, Appel S (2013) In vitro suppression of immune responses using monocyte-derived tolerogenic dendritic cells from patients with primary Sjogren’s syndrome. Arthritis Res Ther 15: R114. 24025795 10.1186/ar4294 PMC3978468

[40] Sparwasser T, Koch E, Vabulas R, Heeg K, Lipford G, et al (1998) Bacterial DNA and immunostimulatory CpG oligonucleotides trigger maturation and activation of murine dendritic cells. Eur J Immunol 28: 2045–2054. 9645386 10.1002/(SICI)1521-4141(199806)28:06<2045::AID-IMMU2045>3.0.CO;2-8

[41] Bauer M, Heeg K, Wagner H, Lipford GB (1999) DNA activates human immune cells through a CpG sequence-dependent manner. Immunology 97: 699–705. 10457226 10.1046/j.1365-2567.1999.00811.x PMC2326885

[42] Harrington LE, Hatton RD, Mangan PR, Turner H, Murphy TL, et al (2005) Interleukin 17-producing CD4+ effector T cells develop via a lineage distinct from the T helper type 1 and 2 lineages. Nat Immunol 6: 1123–1132. 16200070 10.1038/ni1254

[43] Purvis HA, Stoop JN, Mann J, Woods S, Kozijn AE, et al (2010) Low-strength T-cell activation promotes Th17 responses. Blood 116: 4829–4837. 20713963 10.1182/blood-2010-03-272153 PMC3223073

[44] Kantoff PW, Schuetz TJ, Blumenstein BA, Glode LM, Bilhartz DL, et al (2010) Overall survival analysis of a phase II randomized controlled trial of a Poxviral-based PSA-targeted immunotherapy in metastatic castration-resistant prostate cancer. J Clin Oncol 28: 1099–1105. 20100959 10.1200/JCO.2009.25.0597 PMC2834462

[45] Steinman RM, Banchereau J (2007) Taking dendritic cells into medicine. Nature 449: 419–426. 17898760 10.1038/nature06175

[46] Nava S, Dossena M, Pogliani S, Pellegatta S, Antozzi C, et al (2012) An optimized method for manufacturing a clinical scale dendritic cell-based vaccine for the treatment of glioblastoma. PloS One 7: e52301. 23284979 10.1371/journal.pone.0052301 PMC3527532

[47] Carmi Y, Rinott G, Dotan S, Elkabets M, Rider P, et al (2011) Microenvironment-derived IL-1 and IL-17 interact in the control of lung metastasis. J Immunol 186: 3462–3471. 21300825 10.4049/jimmunol.1002901

[48] Zuo JH, Zhu W, Li MY, Li XH, Yi H, et al (2011) Activation of EGFR promotes squamous carcinoma SCC10A cell migration and invasion via inducing EMT-like phenotype change and MMP-9-mediated degradation of E-cadherin. J Cell Biochem 112: 2508–2517. 21557297 10.1002/jcb.23175

[49] Yan L, Borregaard N, Kjeldsen L, Moses MA (2001) The high molecular weight urinary matrix metalloproteinase (MMP) activity is a complex of gelatinase B/MMP-9 and neutrophil gelatinase-associated lipocalin (NGAL). Modulation of MMP-9 activity by NGAL. J Biol Chemi 276: 37258–37265. 10.1074/jbc.M106089200 11486009

[50] Granucci F, Zanoni I, Ricciardi-Castagnoli P (2008) Central role of dendritic cells in the regulation and deregulation of immune responses. Cell Mol Life Sci: CMLS 65: 1683–1697. 18327662 10.1007/s00018-008-8009-2 PMC11131678

[51] Hochrein H, O’Keeffe M, Luft T, Vandenabeele S, Grumont RJ, et al (2000) Interleukin (IL)-4 is a major regulatory cytokine governing bioactive IL-12 production by mouse and human dendritic cells. J Exp Med 192: 823–833. 10993913 10.1084/jem.192.6.823 PMC2193283

[52] Nairz M, Fritsche G, Brunner P, Talasz H, Hantke K, et al (2008) Interferon-gamma limits the availability of iron for intramacrophage Salmonella typhimurium. Eur J Immunol 38: 1923–1936. 18581323 10.1002/eji.200738056

[53] Reilly PT, Teo WL, Low MJ, Amoyo-Brion AA, Dominguez-Brauer C, et al (2013) Lipocalin 2 performs contrasting, location-dependent roles in APCmin tumor initiation and progression. Oncogene 32: 1233–1239. 22614012 10.1038/onc.2012.159 PMC3594828

[54] Hvidberg V, Jacobsen C, Strong RK, Cowland JB, Moestrup SK, et al (2005) The endocytic receptor megalin binds the iron transporting neutrophil-gelatinase-associated lipocalin with high affinity and mediates its cellular uptake. FEBS letters 579: 773–777. 15670845 10.1016/j.febslet.2004.12.031

[55] Yacoub MR, Colombo G, Marcucci F, Caminati M, Sensi L, et al (2012) Effects of sublingual immunotherapy on allergic inflammation: an update. Inflamm Allergy Drug Targets 11: 285–291. 22506880 10.2174/187152812800958988

