# Supplementary figures and images for: Dendritic Cell-Secreted Lipocalin2 Induces CD8+ T-Cell Apoptosis, Contributes to T-Cell Priming and Leads to a TH1 Phenotype
Source: PLoS One. 2014 Jul 10;9(7):e101881. doi: 10.1371/journal.pone.0101881 (PMC4092100; doi:10.1371/journal.pone.0101881)

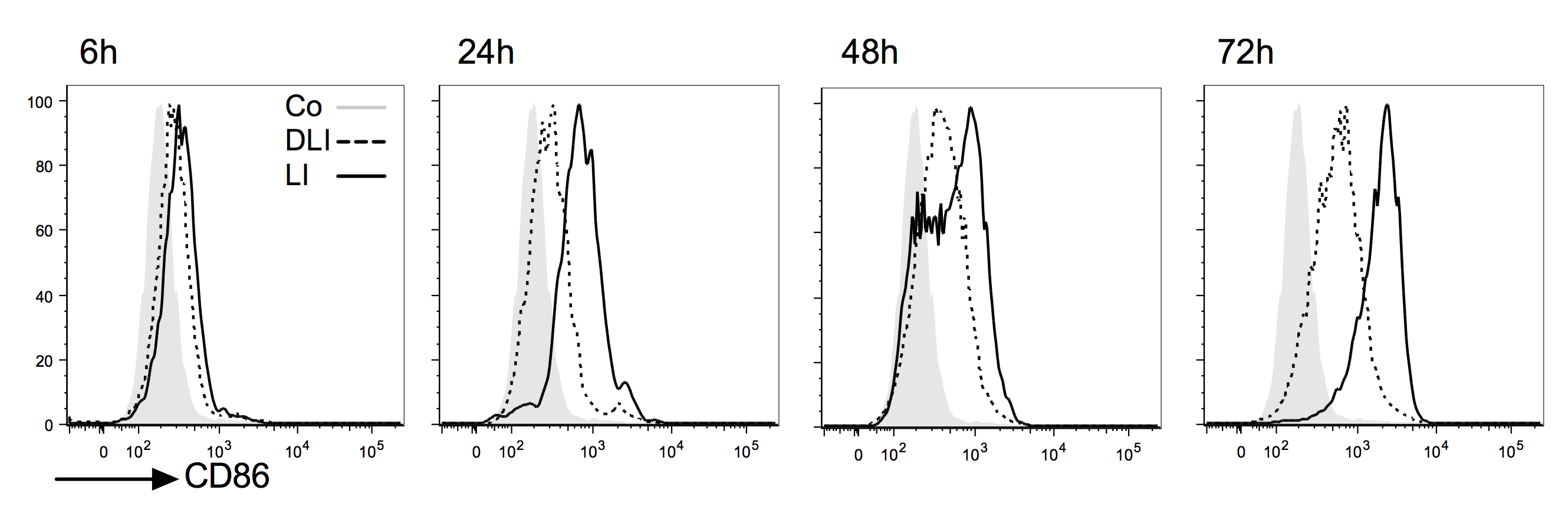

Supplement: Figure S1 — FACS analysis of human monocytes-derived DC maturation after treatment with Dex+LPS+IFN-γ (DLI), LPS+IFN-γ (LI) and the negative control (Co) and stained with CD86. (TIF) [file pone.0101881.s001.tif]

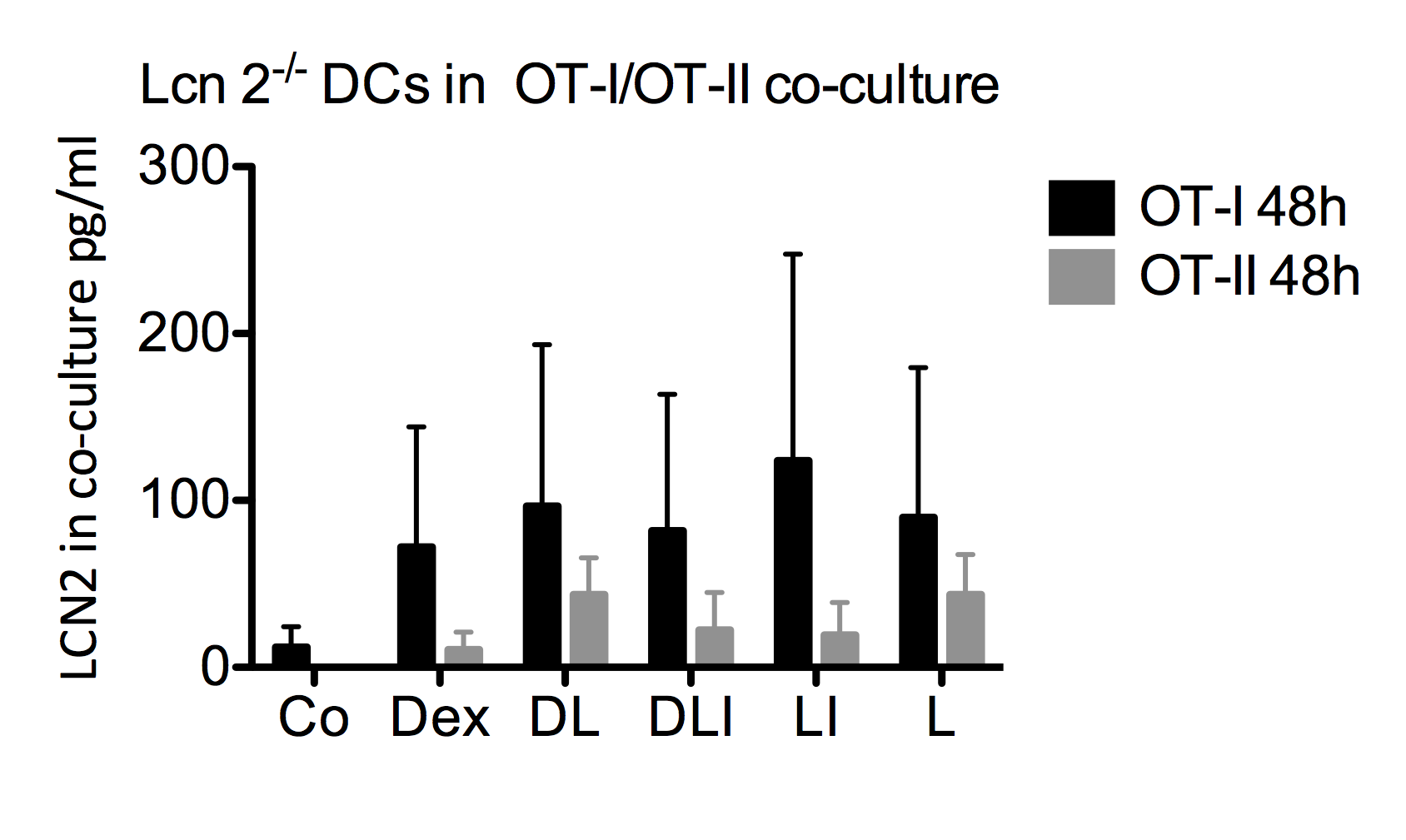

Supplement: Figure S2 — LCN2 expression in Lcn2−/− DC/OT-I and OT-II T-cell co-cultures, the protein amount is calculated in pg/ml, while in WT DC/OT-cell co-cultures it is in ng/ml. (TIF) [file pone.0101881.s002.tif]

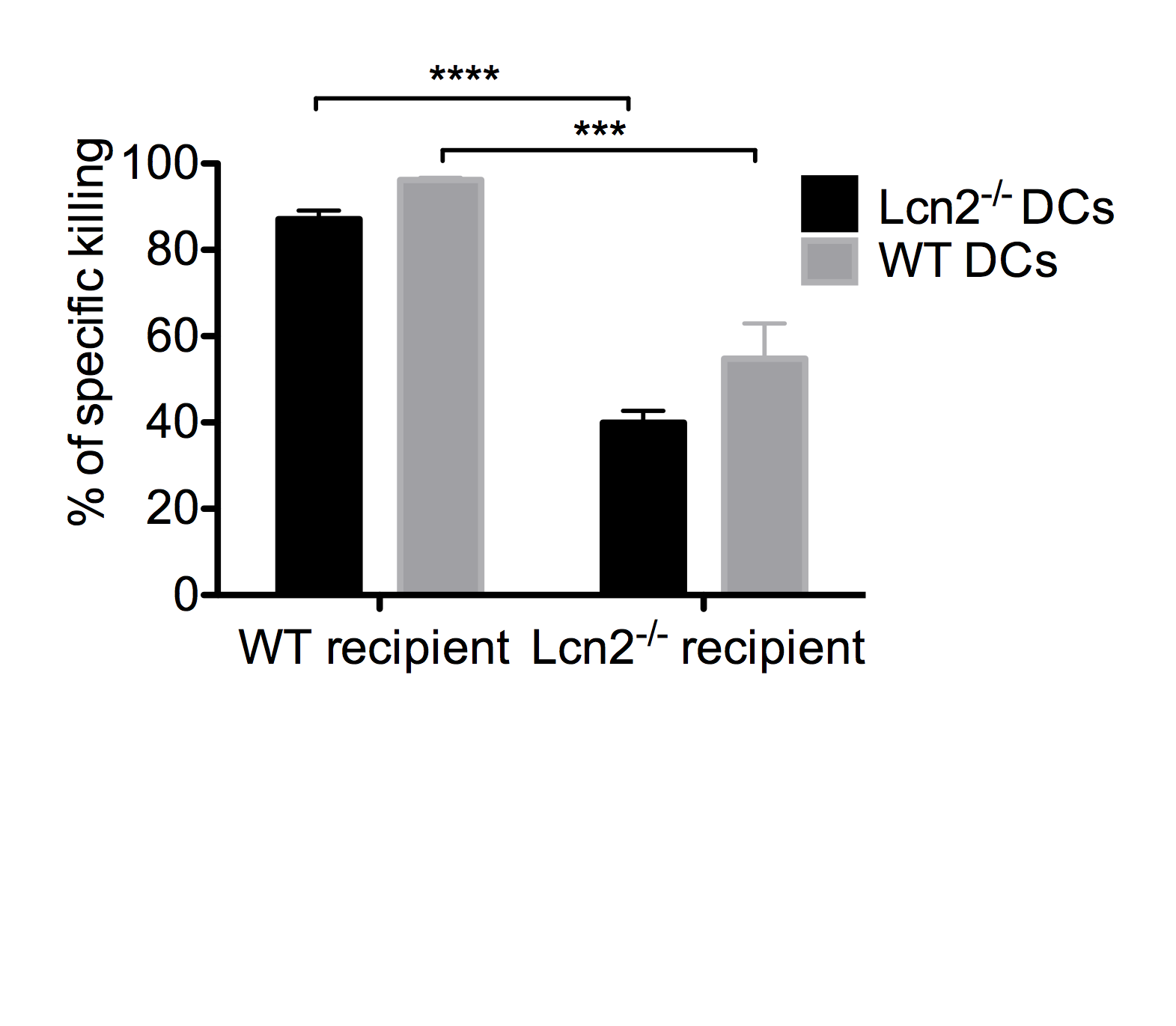

Supplement: Figure S3 — Comparison of the mice killing ability, it is referred to the in vivo CTL assay with DC immunization. (TIF) [file pone.0101881.s003.tif]

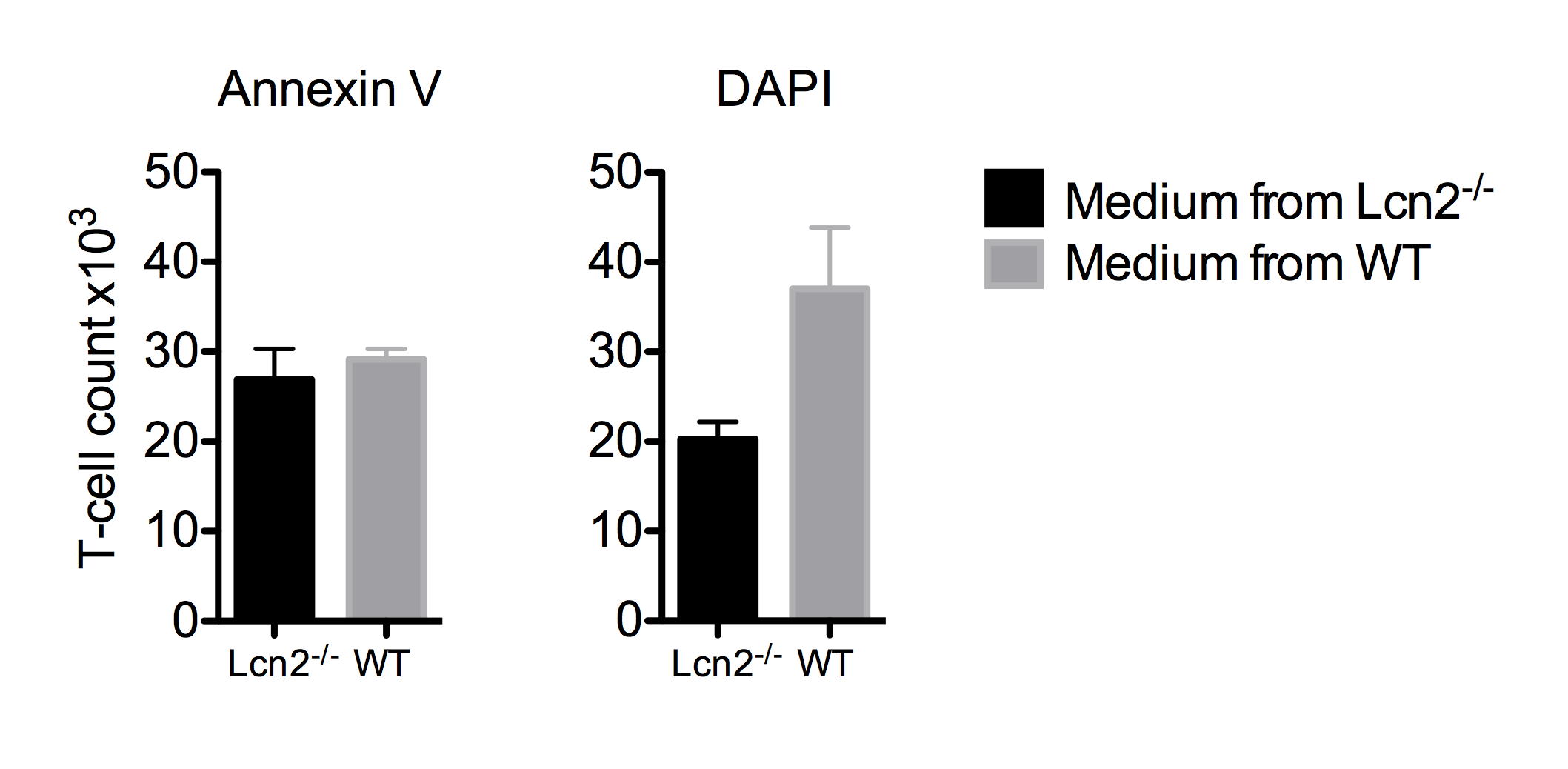

Supplement: Figure S4 — T-cells in culture with conditioned medium from 24 h-LPS-treated WT and Lcn2−/− DCs. (TIF) [file pone.0101881.s004.tif]

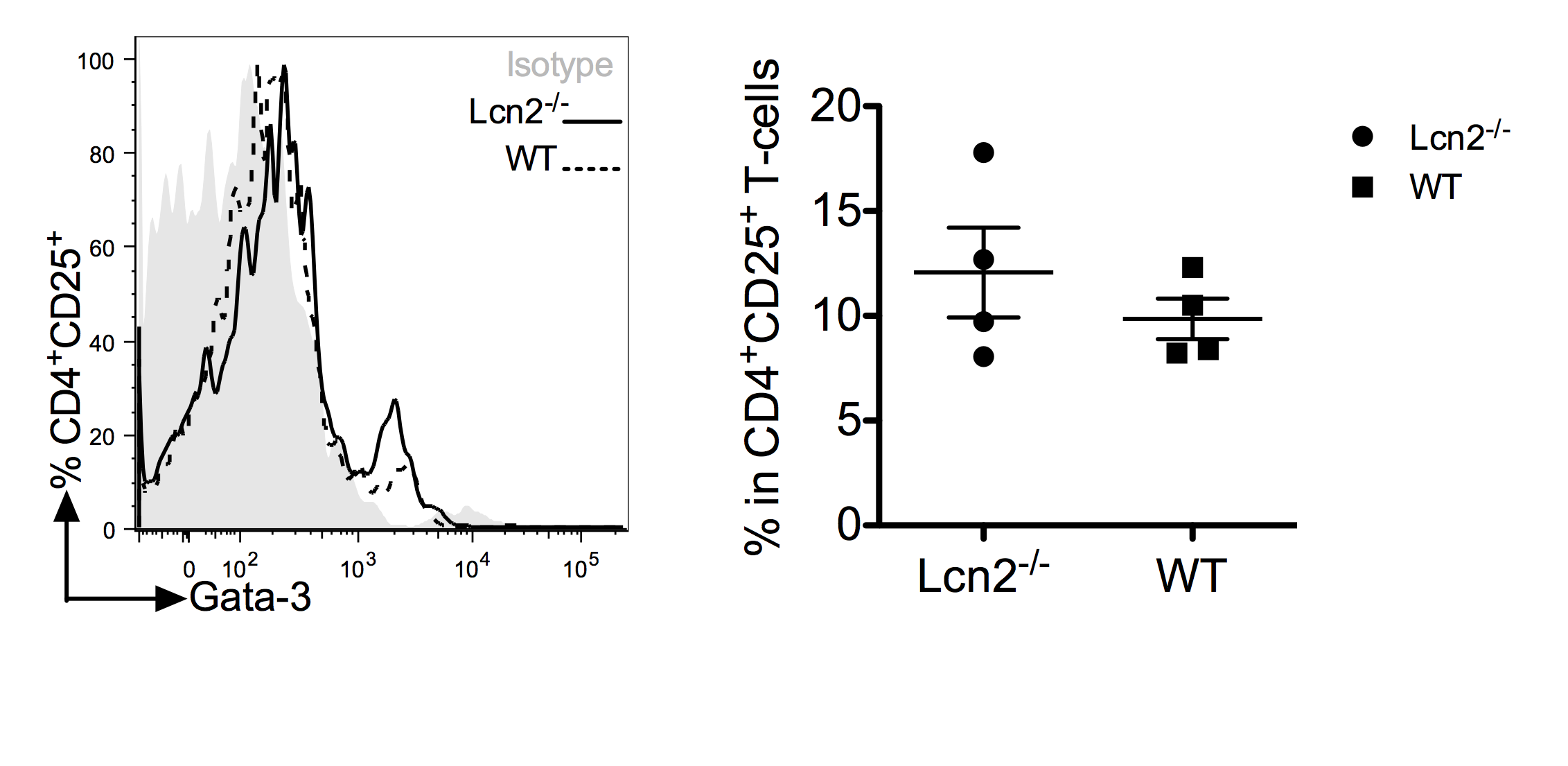

Supplement: Figure S5 — Intracellular staining for the transcription factor T helper 2 (Gata3). (TIF) [file pone.0101881.s005.tif]

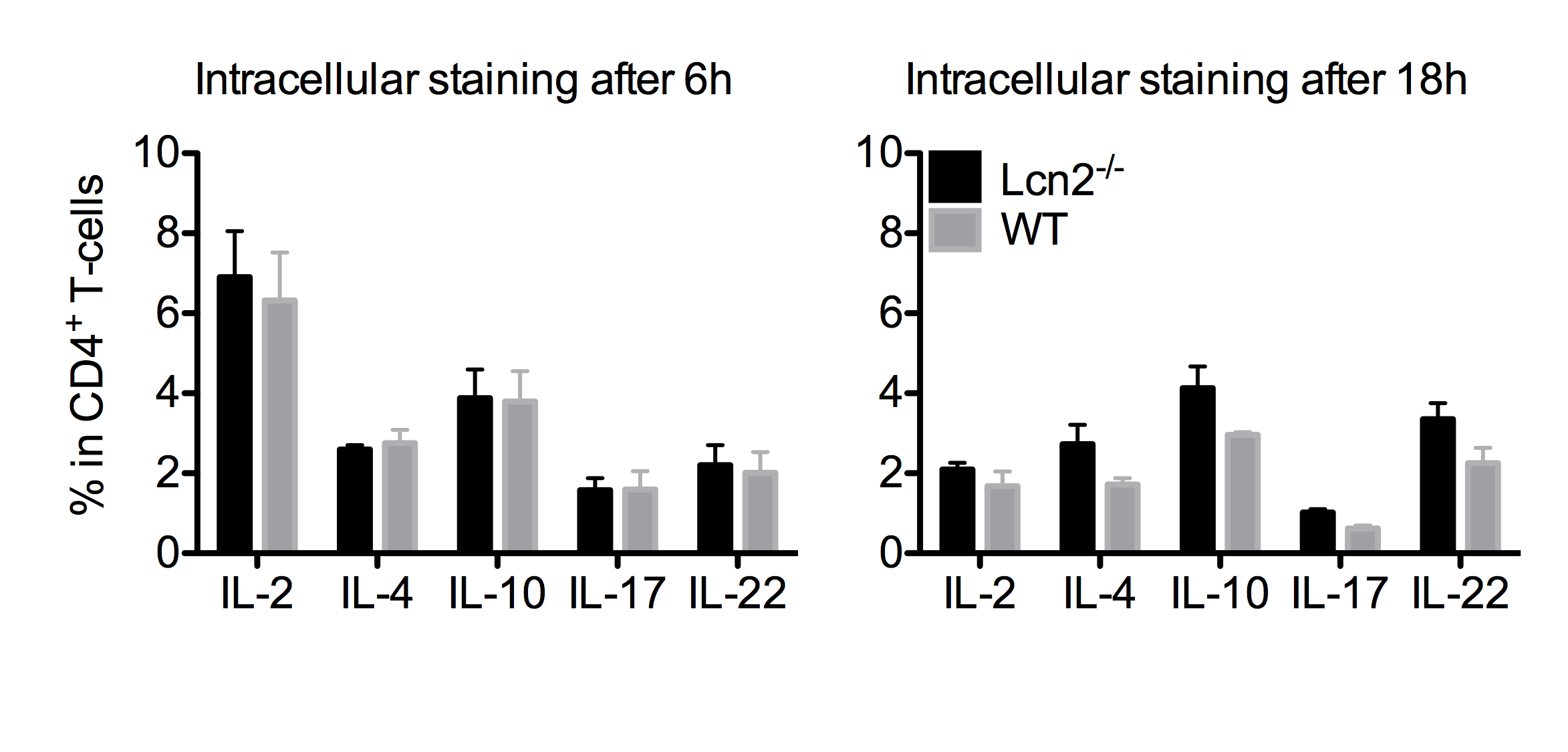

Supplement: Figure S6 — Intracellular staining of cytokines in CD4+ T-cells. (TIF) [file pone.0101881.s006.tif]
